# Colonization of methicillin-resistant Staphylococcus aureus among healthcare students: an integrative review

**DOI:** 10.1590/1516-3180.2020.0564.R2.22042021

**Published:** 2021-11-15

**Authors:** Erika Morganna Neves de Oliveira, Ana Raquel Batista de Carvalho, Adriano Menis Ferreira, Luana Kelle Batista Moura, Andreia Rodrigues Moura da Costa Valle, Daniela Reis Joaquim de Freitas, Maria Eliete Batista Moura

**Affiliations:** I MSc, PhD. Nurse, Nursing Postgraduate Program, Universidade Federal do Piauí (UFPI), Teresina (PI), Brazil.; II MSc. Doctoral Student, Nursing Postgraduate Program, Universidade Federal do Piauí (UFPI), Teresina (PI), Brazil.; III PhD. Nurse and Professor, Nursing Postgraduate Program, Universidade Federal de Mato Grosso do Sul (UFMS), Três Lagoas (MS), Brazil.; IV PhD. Dentist and Professor, Department of Dentistry, Centro Universitário Santo Agostinho, Teresina (PI), Brazil.; V PhD. Nurse and Professor, Nursing Postgraduate Program, Universidade Federal do Piauí (UFPI), Teresina (PI), Brazil.; VI PhD. Biologist and Professor, Department of Microbiology, Universidade Federal do Piauí (UFPI), Teresina (PI), Brazil.; VII PhD. Nurse and Professor, Nursing Postgraduate Program, Universidade Federal do Piauí (UFPI), Teresina (PI), Brazil.

**Keywords:** Students, health occupations, Methicillin resistance, Methicillin-resistant Staphylococcus aureus, Staphylococcus aureus, Students, Multiresistance, Healthcare students, Colonization, Microorganisms

## Abstract

**BACKGROUND:**

Methicillin-resistant *Staphylococcus aureus* (MRSA) infection is a worldwide concern given its presence even in non-hospitalized healthy individuals, such as university students.

**OBJECTIVE:**

To identify in the literature the prevalence of colonization by MRSA among healthcare students.

**DESIGN AND SETTING:**

Integrative review of the literature conducted in Universidade Federal do Piauí.

**METHOD:**

A search for primary studies was performed in the following databases: Medical Literature Analysis and Retrieval System on-line; Cumulative Index to Nursing and Allied Health Literature; Web of Science; Scopus; and LILACS.

**RESULTS:**

This review included 27 studies that demonstrated MRSA infection prevalence ranging from 0.0 to 15.3% among students.

**CONCLUSION:**

The prevalence of colonization of MRSA among healthcare students is high, and the nasal cavity was cited as an important reservoir location for these microorganisms.

## INTRODUCTION

*Staphylococcus aureus* is considered to be a persistent member of the human endogenous microbiota and has historically been associated with important and serious cases of infection. It has the ability to rapidly develop resistance to antibiotics. Methicillin-resistant *Staphylococcus aureus* (MRSA) is considered to be a paradigm for bacterial infections, since it is associated with high rates of morbidity and mortality.^1–3^

In assisting carriers of bacterial infections or colonized or infected patients, or in handling contaminated objects, healthcare workers’ hands can become contaminated. These workers may subsequently transmit the microorganism to other patients. However, this situation is not exclusive to the hospital environment. Clinically manifested diseases in the community or in professionals and/or patients may lead to situations in which some individuals are asymptomatic carriers, also called colonized individuals or simply carriers, when the disease is present in the host organism without causing apparent manifestations.^1,4^ In the United States and Taiwan, the prevalence of strains acquired in the community is 52%, thus exceeding the proportion of strains acquired in hospital environments.^5^ There have also been reports of cases of MRSA acquired in the community.^6,7^

Healthcare students play an important role in the epidemiology and pathogenesis of *Staphylococcus aureus* infection and can act as a source of dissemination both in the community and in hospital environments, and for carrying bacteria from one of these environments to another.^1^

In Brazil, this topic has been little addressed, but it is known that the presence of MRSA among students has been gradually spreading.^1^ Hence, it has become relevant to summarize the knowledge of MRSA that has resulted from research on this subject.

## OBJECTIVE

The objective of this study was to identify in the literature the prevalence of colonization by methicillin-resistant *Staphylococcus aureus* among healthcare students.

## METHODS

### Research design

This study was an integrative review of the literature, incorporating a method of searching for secondary data.^8^ To preserve methodological rigor, the following steps were taken to conduct this review: formulation of the research question; idealization of sampling plan and data collection strategies; extraction of relevant data from studies included in the review; and, finally, analysis and interpretation of the data.^8^

The research question was elaborated in accordance with the PVO strategy (P – population; V – variable of interest; O – outcome). Thus, in line with the objective of the study, the following structure was used: P - healthcare students; V – methicillin-resistant *Staphylococcus*; O – prevalence.^9^ Therefore, the following question was asked: “What evidence is available in the literature regarding the prevalence of methicillin-resistant *Staphylococcus aureus* colonization among healthcare students?”

### Data collection period

Searching for and selection of studies took place between the months of November 2019 and January 2020 and were carried out by two independent reviewers. Any divergences were resolved by a third reviewer.

### Selection criteria

After the search stage, original articles were selected, based on reviewing their titles and abstracts, in accordance with the following inclusion criteria: original articles covering the population of undergraduate students in the field of healthcare who experienced clinical activities that brought them into direct contact with patients.

The full text of each article was then read, with a view to choosing studies that answered the research question. Through this process, articles involving high school or technical students, those that did not comply with selection criteria mentioned above, those that did not answer the research question and those that were duplicates were excluded, as also were opinion articles, theoretical reflections, dissertations and book chapters.

### Data collection

The following databases were selected: Medical Literature Analysis and Retrieval System online (MEDLINE) via National Library of Medicine National Institutes of Health (PubMed); Cumulative Index to Nursing and Allied Health Literature (CINAHL); Web of Science; Scopus; and Literatura Latino-americana e do Caribe em Ciências da Saúde (LILACS) via Biblioteca Virtual em Saúde (BVS).

The descriptors and keywords used in the search were applied in accordance with particularities of each database. They were obtained by consulting the Descritores em Ciências da Saúde (DeCS), Medical Subject Headings (MeSH) and titles from CINAHL. During the search, descriptors were cross-referenced with each other using the Boolean operators “or” and “and”. Descriptors were inserted in the English language, since all journals indexed in these databases have descriptors in English in their articles; with the exception of BVS, in which descriptors were inserted in English and Portuguese. To expand the search, there was no limitation on the time of publication or language. Table 1 shows the descriptors used in this study and summarizes how the search was carried out.

**Table 1:** Descriptors used in the search strategy for primary articles. Teresina (PI), Brazil, 2020

Data Source	
Descriptors and Keywords	
**BVS**	
	Estudantes OR Estudantes de Ciências da Saúde OR Estudantes de Enfermagem OR Estudantes de Farmácia OR Estudantes de Medicina OR Aluno OR Alunos OR Estudante OR Enfermeiras Estudantes OR Alunos de Enfermagem OR Estudante de Enfermagem OR Enfermeiros Estudantes
	Staphylococcus aureus
	Resistência à meticilina
**PubMed/ WEB OF SCIENCE/ SCOPUS**	
	“Students” OR “Students, Health Occupations” OR “Students, Nursing” OR “Students, Pharmacy” OR “Students, Medical” OR Students, Dental” OR “Health Occupations Students” OR “Health Occupations Student” OR “Student, Nursing” OR “Nursing Student” OR “Nursing Students” OR “Pharmacy Students” OR “Student, Pharmacy” OR “Pharmacy Student” OR “Medical Students” OR “Student, Medical” OR “Medical Student” OR “Dental Students” OR “Student, Dental” OR “Dental Student”
	“Staphylococcus aureus”
	“Methicillin Resistance” OR “Resistance, Methicillin” OR “Methicillin-Resistant” OR “Methicillin Resistant”
**CINAHL**	
	Students, Health Occupations
	Staphylococcus aureus
	Methicillin-Resistant Staphylococcus Aureus

### Data processing and analysis

**Figure 1:**
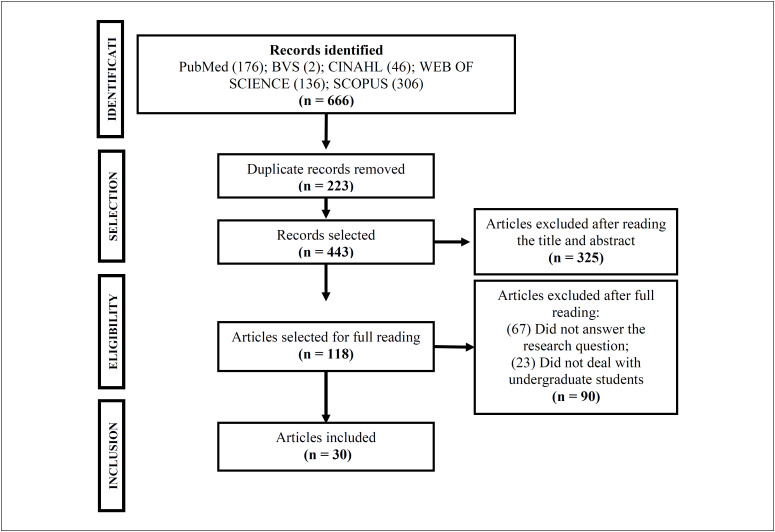
Study selection flowchart. Teresina (PI), Brazil, 2020.

The studies thus found were exported to the Endnote reference manager software, version 20 (Clarivate Analytics, Philadelphia, United States), in order to identify duplicates and gather together all publications. In addition, the reference lists of these articles were consulted in order to find any additional studies. The selection of studies followed the recommendations of the Preferred Reporting Items for Systematic Reviews and Meta-Analyses (PRISMA)^9^ (Figure 1).

For data analysis and extraction, a data collection instrument that had been validated by Ursi was chosen for this study and was adapted for use in it.^10^ Furthermore, the protocol for this review was previously assessed by experts in the method used. After fully evaluating the texts, a descriptive analysis on the results found was carried out, in which a synthesis of all the studies included in the review was presented, along with comparisons between them.

## RESULTS

The final sample for this review comprised 30 primary articles, which were characterized taking into account the authors, year of publication, country, objective and main results (Table 2). These studies were published in the years 2010, 2012, 2013, 2014, 2015, 2016, 2017, 2018 and 2019.^3,5,7,11–37^

**Table 2:** Characterization of studies included in this review (n = 30). Teresina (PI), Brazil, 2020

Article	Authors/Year	Location	Objective	Site
A1	Prates et al.^11^ 2010	Brazil	To determine the prevalence of nasal transportation of *S. aureus* in university students.	Nostrils
A2	Syafinaz et al.^12^ 2012	Malaysia	To determine the prevalence of *S. aureus* nasal carriers among medical students.	Nostrils
A3	Bettin et al.^13^ 2012	Colombia	To investigate the nasal transportation of Panton-Valentin leukocidin-positive *S. aureus* strains, categories of transportation and risk factors associated with colonization, in medical students.	Nostrils
A4	Chen et al.^14^ 2012	China	To investigate whether clinical exposure in the hospital affects MRSA nasal transportation among medical students.	Nostrils
A5	Sabri et al.^16^ 2013	Palestine	To investigate the prevalence of nasal transportation of *S. aureus* and MRSA.	Nostrils
A6	López-Aguilera et al.^15^ 2013	Spain	To determine the prevalence of nasal carriers of sensitive and methicillin-resistant *S. aureus* and evaluate knowledge of and adherence to hand hygiene among students.	Nostrils
A7	Mat Azis et al.^17^ 2014	Malaysia	To evaluate the transportation of *S. aureus* and its persistence in students of health sciences.	Nostrils
A8	Malik et al.^18^ 2014	Brunei	To determine the prevalence of the status of nasal carrier of *S. aureus* and MRSA among healthy young people.	Nostrils
A9	Krishnamurthy et al.^19^ 2014	India	To examine the influence of exposure to the hospital environment on MRSA transportation, MRSA antimicrobial resistance patterns and presence of genes that encode five determinants of extracellular pathogenicity.	Nostrils, throat and hand palms
A10	Demirel et al.^20^ 2014	Turkey	To investigate the prevalence of methicillin-sensitive (CA-MSSA) and resistant (CA-MRSA) *S. aureus*, including inducible sleepers (ID-MRSA), in *S. aureus* and MRSA strain genotypes from nasal cultures.	Nostrils
A11	Renushri et al.^21^ 2014	India	To assess the influence of exposure to the hospital environment on MRSA transportation.	Nostrils and throat
A12	Ribeiro et al.^22^ 2014	Brazil	To identify *S. aureus* and MRSA in university students.	Nostrils and palm hands
A13	Holý et al.^24^ 2015	Czech Republic	To investigate the prevalence of nasal transportation of *S. aureus* and MRSA in healthy people aged 18–26 years. To find out whether the prevalence of nasal transportation strains of *S. aureus* and MRSA varies over the years of studies. To compare general medical students from year 1 and year 5 for nasal transportation of *S. aureus* and MRSA strains.	Nostrils
A14	Zakai et al.^23^ 2015	Saudi Arabia	To identify MRSA nasal carrier status among medical students during their clinical rotations.	Nostrils
A15	Collazos Marín et al.^25^ 2015	Colombia	To establish the genetic diversity of *S. aureus* isolates and detect the presence of mecA gene in isolated strains in asymptomatic medical students who were in their clinical rotation phase in a hospital.	Nostrils
A16	Petti et al.^26^ 2015	Italy	To evaluate the MRSA carrier rate in a sample of dental students.	Nostrils, throat and palm hands
A17	Hogan et al.^3^ 2016	Madagascar	To examine the prevalence and clonal epidemiology of nasal *S. aureus* and MRSA among healthcare professionals and non-medical university students.	Nostrils
A18	Javaeed et al.^27^ 2016	Pakistan	To assess the prevalence of MRSA transportation in healthy medical students.	Nostrils
A19	Subri et al.^28^ 2016	Malaysia	To determine the prevalence of nasal colonization of *S. aureus* and its susceptibility to antibiotics among pre-clinical and clinical physicians and nursing students.	Nostrils
A20	Ansari et al.^29^ 2016	Nepal	To evaluate the rate of nasal colonization of *S. aureus*, its methicillin-resistant strains and risk factors in medical students before clinical exposure.	Nostrils
A21	Okamo et al.^30^ 2016	Tanzania	To determine the prevalence of *S. aureus* and MRSA nasal transportation among medical students, and the antimicrobial susceptibility of isolated profiles of *S. aureus*, and to verify the association of *S. aureus* nasal transportation with demographic and clinical characteristics.	Nostrils
A22	Baek et al.^31^ 2016	South Korea	To determine the prevalence rate of nasal colonization by MRSA among dental students and identify the characteristics of the strains isolated.	Nostrils
A23	Radhakrishna et al.^32^ 2016	India	To establish the prevalence and pattern of S. aureus antibiograms, with special emphasis on MRSA among students of the second year.	Nostrils
A24	Abroo et al.^7^ 2017	Iran	To investigate the prevalence, antimicrobial susceptibility and molecular factors characteristic of CA (community acquired) MRSA among two groups of college students (medical and non-medical).	Nostrils
A25	Budri et al.^33^ 2018	Ireland	To investigate co-located nasal *Staphylococcus aureus* and coagulase-negative staphylococci (CoNS), recovered from healthy medical students in a preclinical year and the transportation of genes and common elements to both species that may contribute to the evolution of *S. aureus* and MRSA.	Nostrils
A26	Al-Tamimi et al.^34^ 2018	Jordan	To investigate the prevalence, standard antimicrobial susceptibility, antibiotic resistance genes and risk factors of medical students with MRSA.	Nostrils
A27	Suhaili et al.^35^ 2018	Malaysia	To evaluate the antimicrobial susceptibility profile of *S. aureus* strains isolated from university students and to determine the prevalence of resistance to constitutive and inducible clindamycin, in which the latter would be capable of causing therapeutic failure due to false in vitro susceptibility to clindamycin.	Nostrils
A28	Onanuga et al.^36^ 2019	Nigeria	To determine the antibiogram and the virulent characteristics of nasal *S. aureus*, accessing its profile of resistance to antibiotics and potential pathogens in healthy students at the University of the Niger Delta, Bayelsa State, Nigeria.	Nostrils
A29	Szymanek-Majchrzak et al.^37^ 2019	Poland	To evaluate and compare the level of colonization of *S. aureus* (MRSA or MSSA) among medical students and evaluate the sensitivity of the strains.	Nostrils
A30	Efa et al.^5^ 2019	Ethiopia	To determine the nasal transportation of MRSA and its antimicrobial susceptibility patterns among medical students at the Jimma University Medical Center (JUMC), southwestern Ethiopia.	Nostrils

Regarding the locations of the studies, they were carried out in Brazil, Malaysia, Colombia, China, Palestine, Spain, Brunei, India, Turkey, Czech Republic, Saudi Arabia, Madagascar, Pakistan, Nepal, Tanzania, South Korea, Iran, Ireland, Jordan, Italy, Nigeria, Poland and Ethiopia.^3,5,7,11,12,14–22,24–37^

The populations addressed by the researchers of these 30 studies were nursing students, medical students, health science students and dental students.^3,5,7,12–18,20,21,23–25,27,29–30,33–37^ Two studies involved students from more than one undergraduate course.^11,22,28^

To detect colonizing microorganisms, samples were collected using the technique of swab smears from nasal specimens, in all of these studies except for four studies, in which specimens were collected from more than one anatomical site.^3,5,7,15–17,18–27–30,33–37^

Regarding the prevalence of MRSA, the student population in some studies was divided into groups before exposure to healthcare and after such exposure.^14,19,21,23,26^ The percentages found are shown in Table 3.

**Table 3:** Prevalence of methicillin-resistant *Staphylococcus aureus*, according to the studies included in this review

	General students	Students before clinical exposure	Students after clinical exposure
A1	2.4%	–	–
A2	0.0%	–	–
A3	1.61%	–	–
A4	2.2%	1.9%	2.4%
A5	9.0%	–	–
A6	2.1%	–	–
A7	3.3%	–	–
A8	–	0.0%	–
A9	6.8 %	4.0%	9.0%
A10	3.0%	–	–
A11	8.2%	4.0%	11.8%
A12	1.9%	–	–
A13	0.0%	–	–
A14	15.3%	0.0%	6.7%
A15	14.3%	–	–
A16	3.2%	3.1%	0.0%
A17	1.3%	–	–
A18	5.5%	–	–
A19	0.0%	–	–
A20	–	4.0%	–
A21	0.3%	0.0%	0.3%
A22	3.1%	–	3.1%
A23	6.1%	–	–
A24	13.1%	–	–
A25	2.1%	2.1%	–
A26	4.1%	–	–
A27	8%	–	–
A28	7.1%	–	–
A29	0.1%	–	–
A30	8.4%	–	–

## DISCUSSION

Worldwide, occurrence of healthcare-associated infections (HAI) is one of the main public health problems, with severe human and economic repercussions. According to the Centers for Disease Control and Prevention (CDC), MRSA infections have outperformed HIV as the leading cause of morbidity and mortality in the United States.^38^

Studies have revealed high prevalence of MRSA in patients and healthcare professionals with exposure to the healthcare system.^23,38,39^ However, the results systematized in the present study revealed that presence of MRSA has also been reported among non-hospitalized healthy individuals, such as undergraduate students, ranging from 0.0% to 15.3%.^14,25,26^

Data in the literature have highlighted occurrences of MRSA infection in healthy populations that live in agglomerations or experience such conditions but which have little or no contact with healthcare services, as is the case of undergraduate students within the field of healthcare.^1,40^ This was observed in the present study, thus indicating that MRSA infection was present in students who were not exposed to hospital environments. This may indicate the presence of community-acquired MRSA strains.^1^ It needs to be borne in mind that in the studies discussed here, students who had been hospitalized within the last few months had been excluded, considering that hospitalization could be a confounding factor for occurrences of MRSA.

Identification of high frequencies of MRSA in students before they were exposed to experiences of clinical care is a matter for concern. It indicates that there is a need for effective infection prevention and control policies, in relation to hygiene and surveillance.^5^

Clinical practice among students in the field of healthcare is part of the teaching-learning process. In relation to this process, there is exposure to occupational risks, especially through recognition of the variability of care provided to patients.^1,5^ In this regard, studies that have addressed the prevalence of MRSA among students after exposure to hospital environments can provide evidence that exposure to MRSA in hospitals can play a critical role in achieving nasal colonization by MRSA.

According to the literature, the nostrils are the main colonization site for *Staphylococcus aureus*, whose prevalence reaches, on average, 40% in the adult population.^1,5–6^ Possibly for this reason, the nasal cavity was the site most chosen by researchers for sample collection in their studies, thus showing the importance of the upper airways in transmission and acquisition of pathogenic microorganisms. The throat and palms are also important reservoirs for MRSA.^19,21,22,26^

It is known that students in the field of healthcare, as they progress through the curriculum with increasing complexity of care practices, whether in hospitals or other healthcare delivery environments, become carriers of microbes. In this, acquisition of *Staphylococcus aureus* is considered to be a major concern, especially with regard to MRSA.^1^

Thus, MRSA rates in students may increase according to their clinical exposure, as well as from isolated occurrences. In another study, there was greater potential for virulence in samples from groups working in clinics.^5^ This aspect of infection could not be analyzed in the present study, since the studies included in this review were cross-sectional, which did not allow the study sample to be monitored.

The prevalences found need to be analyzed with caution, considering that occurrences of infections caused by MRSA may differ according to the scenarios within which they occur. This may be due to measures that are taken to control infection and may be dependent on effective implementation.^5^ Likewise, the MRSA rate also varies in different locations.^27,41^

This study presented some limitations due to the choice of databases and keywords. Use of the CINAHL database may have restricted the search, as it is a specific database for the field of nursing. In addition, the choice of databases and keywords may have camouflaged studies on the same topic that were not indexed in the same database. Hence, it can be suggested that similar investigations should be conducted, with cross-referencing of other databases, in order to investigate Brazilian scientific production on colonization by *Staphylococcus aureus* among healthcare students.

## CONCLUSION

The prevalence of colonization by methicillin-resistant *Staphylococcus aureus* among healthcare students is high, and the nasal cavity was cited in this study as an important reservoir for these microorganisms.

Efforts need to be made to implement standards and routines that are designed to limit the spread of MRSA strains among students, given that once MRSA has become established within a community, its eradication and control is difficult. Furthermore, in view of the high morbidity and mortality and exponential growth of series of microbial resistance, implementation of control strategies is prudent.

Therefore, education on infection control measures in undergraduate healthcare courses is of great importance, as also is implementation of adequate and effective infection control programs to reduce the prevalence of MRSA.
